# Sarcomatoid carcinoma of the pancreas with rare long-term survival: a case report

**DOI:** 10.1186/s12957-020-01879-8

**Published:** 2020-05-25

**Authors:** Toshihisa Kimura, Daisuke Fujimoto, Tamotsu Togawa, Makoto Ishida, Atsushi Iida, Yasunori Sato, Takanori Goi

**Affiliations:** 1 Department of Surgery, National Hospital Organization, Tsuruga Medical Center, 33-1, Sakuragaoka, Tsuruga, Fukui, 914-0195 Japan; 2 First Department of Surgery, Faculty of Medicine, University of Fukui, 23-3, Matsuoka Shimoaizuki, Eiheiji-cho, Yoshida-gun, Fukui, 910-1193 Japan; 3 Department of Surgery, Tannan Regional Medical Center, 1-2-31, Saburoku-cho, Sabae, Fukui, 916-8515 Japan; 4 Department of Human Pathology, Kanazawa University Graduate School of Medicine, 13-1, Takara-machi, Kanazawa, Ishikawa 920-8640 Japan

**Keywords:** Sarcomatoid carcinoma, Pancreatic cancer, Long-term survival, Epithelial-mesenchymal transition

## Abstract

**Background:**

Sarcomatoid carcinoma of the pancreas (SCP) tends to have similar or even worse prognosis than that of conventional pancreatic ductal adenocarcinoma. The clinical and pathological features of SCP remain poorly characterized owing to its rarity.

**Case presentation:**

A 58-year-old man was admitted to our hospital with the chief complaints of upper abdominal pain and weight loss. Abdominal contrast computed tomography revealed a 5-cm low-density mass in the pancreatic body. Magnetic resonance cholangiopancreatography revealed an obstruction of the main pancreatic duct and a dilation of the distal main pancreatic duct. Based on the clinicoradiological findings, pancreatic body cancer was suspected, and the distal pancreatectomy was performed. A pathological examination revealed that the tumor was composed of an area of invasive ductal adenocarcinoma and an area of sarcomatous spindle-shaped cells; the latter component predominated. The spindle cells were immunohistochemically positive for both cytokeratin and vimentin, and thus, a pathological diagnosis of SCP was made. In addition, immunohistochemical analysis suggested the sarcomatous component might be derived from the adenocarcinoma component via the process of epithelial-mesenchymal transition. After the operation, the patient received 6 months of chemotherapy with gemcitabine. At 10 years after the operation, the patient is alive with no recurrence.

**Conclusions:**

The current case study presented a SCP patient with long-term survival after the operation. It was worth noting that the sarcomatous component of the tumor pathologically showed lower MIB-1 labeling index compared with those in previously reported SCP cases, which might account for the long-term survival of the patient.

## Background

Sarcomatoid carcinoma is an aggressive malignancy that has both epithelial and mesenchymal features. It is histologically characterized by an admixture of carcinomatous and sarcomatous components. Immunostaining shows that both components express epithelial markers such as cytokeratin, and the sarcomatous component also expresses mesenchymal markers such as vimentin [1].

Sarcomatoid carcinoma primarily occurs in the lungs, esophagus, breast, larynx, and genitourinary tract [2, 3]. Sarcomatoid carcinoma of the pancreas (SCP) is extremely rare, and only a small number of cases have been reported in the English literature [2–12]. Sarcomatoid carcinoma is generally thought to represent a process of epithelial-mesenchymal transition (EMT) of an epithelial tumor, and EMT is a plausible mechanism of tumorigenesis of SCP [3, 11]. SCP is composed of cells with spindle cell morphology, with or without an epithelial/glandular component [1]. On occasion, histological transition can be encountered between the epithelial/glandular component and spindle cells.

It is well established that transforming growth factor-β (TGF-β) induces EMT. The expression of phosphorylated Smad2/3 (pSmad2/3) is regarded as a marker of the occurrence of intracellular signal transduction via TGF-β, and Snail is one of the major transcription factors involved in the regulation of TGF-β-mediated EMT [13]. Fibronectin can serve as an indicator of the occurrence of EMT, where details on the expression of these molecules in SCP remain unknown [14, 15].

The prognosis of SCP tends to be similar to or even worse than that of conventional pancreatic ductal adenocarcinoma [2–12]. Herein, we report a rare case of SCP with long-term survival after the operation.

## Case presentation

A 58-year-old Japanese man with upper abdominal pain and loss of 4 kg in weight over the course of 1 month was referred to our hospital for the examination of a pancreatic mass that had been identified by a previous doctor on abdominal ultrasonography. Laboratory data revealed that the complete blood counts, liver function tests, and amylase and lipase levels were all within the normal range. Elevated fasting blood glucose (152 mg/dl) and HbA1c (5.9%) levels indicated abnormal glucose tolerance. The levels of tumor markers such as carcinoembryonic antigen, carbohydrate antigen 19-9, and Dupan-2 were all within the normal range.

**Fig. 1:**
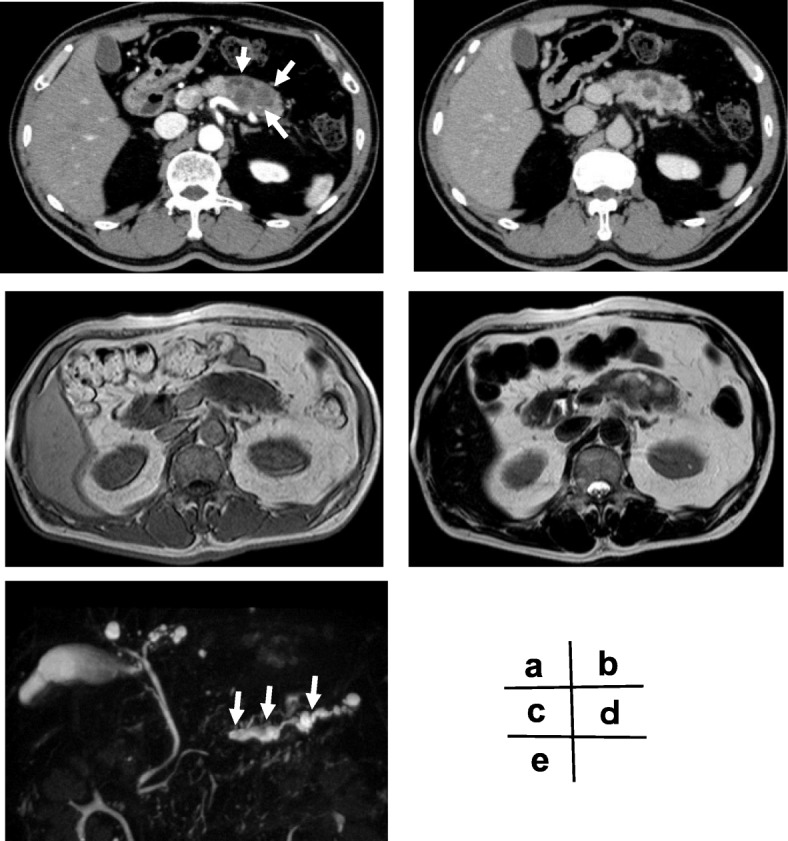
Contrast-enhanced CT scan (early phase) showed a low-density mass measuring 5 cm in diameter in the pancreatic body (arrows) (**a**). Contrast-enhanced CT scan (late phase) (**b**). MRI showed a tumor in the pancreatic body showing low intensity on T1-weighted images (**c**) and relatively high intensity on T2-weighted images (**d**). MRCP revealed a dilation of the distal main pancreatic duct (arrows) (**e**)

A computed tomography (CT) scan showed that the original lesion in the pancreatic body was a complex heterogeneous mass measuring 5.0 cm in diameter that contained cystic and mixed solid areas (Fig. 1a, b). No evidence of metastasis was observed. Magnetic resonance imaging (MRI) revealed a tumor in the pancreatic body that was visualized as low intensity on T1-weighted images (Fig. 1c) and relatively high intensity on T2-weighted images (Fig. 1d). Magnetic resonance cholangiopancreatography (MRCP) revealed an obstruction of the main pancreatic duct and a dilation of the distal main pancreatic duct (Fig. 1e). Based on the diagnosis of pancreatic body cancer, distal pancreatectomy with splenectomy was performed, and regional lymph nodes were removed.

**Fig. 2:**
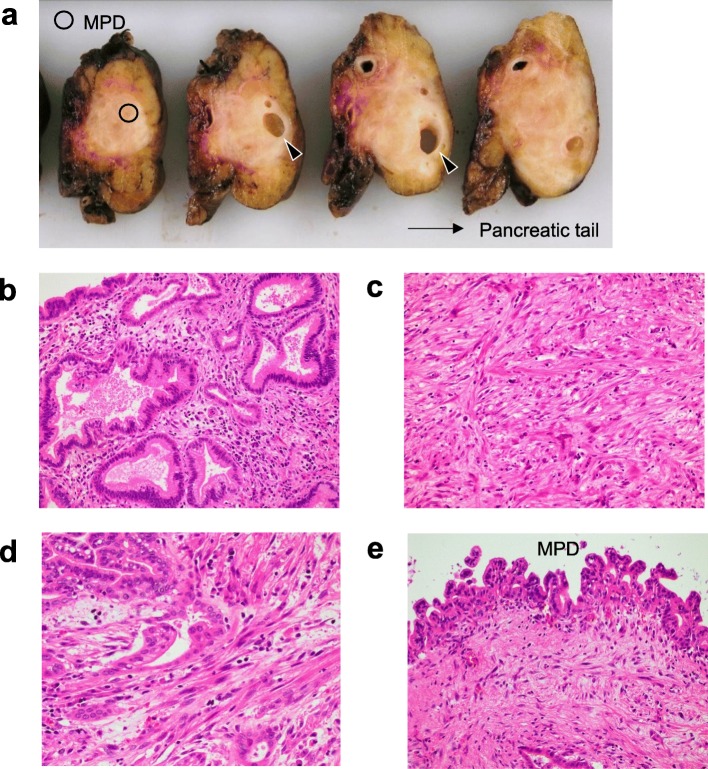
Pathological findings. An ill-defined infiltrative tumor was grossly observed in the pancreatic body (**a**). Histologically, the tumor was composed an area of adenocarcinoma (**b**) and sarcomatous spindle cells (**c**). A transition was observed between the adenocarcinoma component and the sarcomatous component (**d**). The lumen of the main pancreatic duct (MPD) within the tumor was covered by high-grade pancreatic intraepithelial neoplasia (**e**). Arrowheads indicate retention cysts

In the resected specimen, an ill-defined infiltrative tumor was macroscopically observed at the cut surface of the pancreatic body (Fig. 2a). The main pancreatic duct was identifiable only in the portion of the pancreatic head side of the tumor. The cystic area within the tumor corresponded to a retention cyst (Fig. 2a, arrowheads).

Histologically, the tumor was composed of an area of invasive adenocarcinoma and an area of sarcomatous spindle cells (Fig. 2b, c). The vast majority of the tumor was composed of sarcomatous spindle cells, and the adenocarcinoma component accounted for less than 5% of the total tumor. A transition was observed between the adenocarcinoma component and the sarcomatous component (Fig. 2d). The lumen of the main pancreatic duct within the lesion was covered by dysplastic epithelia corresponding to high-grade pancreatic intraepithelial neoplasia (Fig. 2e), and there was no pathological evidence showing that the adenocarcinoma or sarcomatoid component had arisen from intraductal papillary mucinous neoplasm.

The adenocarcinoma component was accompanied by mild lymphocyte infiltration in the tumor stroma, and lymphocyte infiltration was negligible in the sarcomatous component (Fig. 2b, c). The cellularity of the sarcomatous component was not as pronounced, and it contained an abundant fibrous stroma (Fig. 2c). The sarcomatous component invaded the splenic artery and the splenic vein located at the periphery of the tumor. Necrotic tendency, heterologous elements such as bone and cartilage, and osteoclast-like giant cells were not observed.

**Fig. 3:**
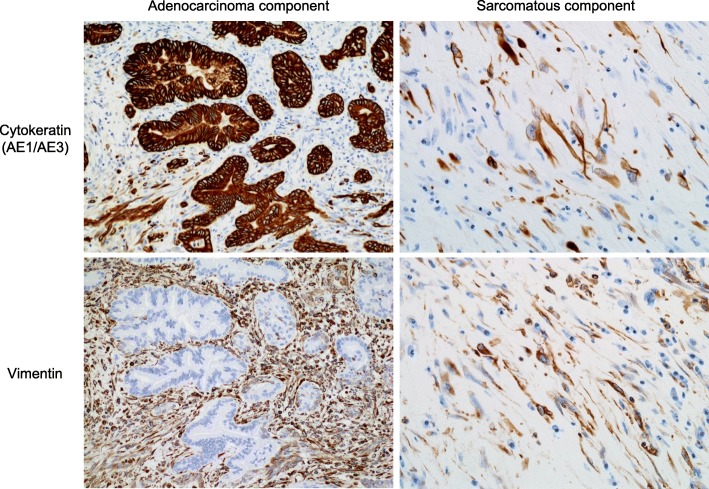
Immunohistochemical findings. The adenocarcinoma component was reactive to an antibody against cytokeratin (AE1/AE3). The sarcomatous component was reactive to antibodies against both cytokeratin and vimentin

**Fig. 4:**
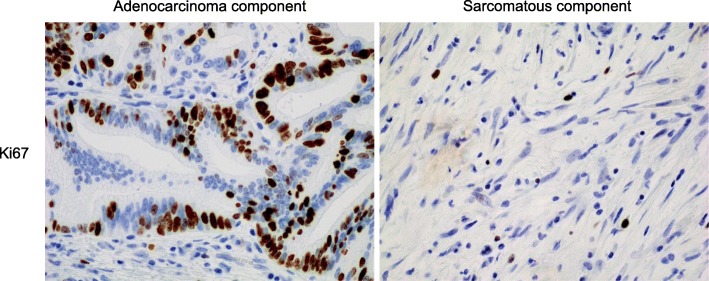
Immunostaining of Ki67. The MIB-1 labeling index was 38% in the adenocarcinoma component and 11% in the sarcomatous component

Immunohistochemically, the adenocarcinoma component was reactive to an antibody against cytokeratin (AE1/AE3), and the sarcomatous component was reactive to antibodies against both cytokeratin and vimentin (Fig. 3). Based on the classification of tumors by the World Health Organization (WHO) (5th edition, 2019), a pathological diagnosis of SCP was made [1]. The MIB-1 labeling index, that was assessed for more than 500 cells at the hot spot, was 38% in the adenocarcinoma component and 11% in the sarcomatous component (Fig. 4). The immunohistochemical expression of programmed death-ligand 1 (PD-L1) was not observed in the adenocarcinoma and sarcomatous components (data not shown).

**Fig. 5:**
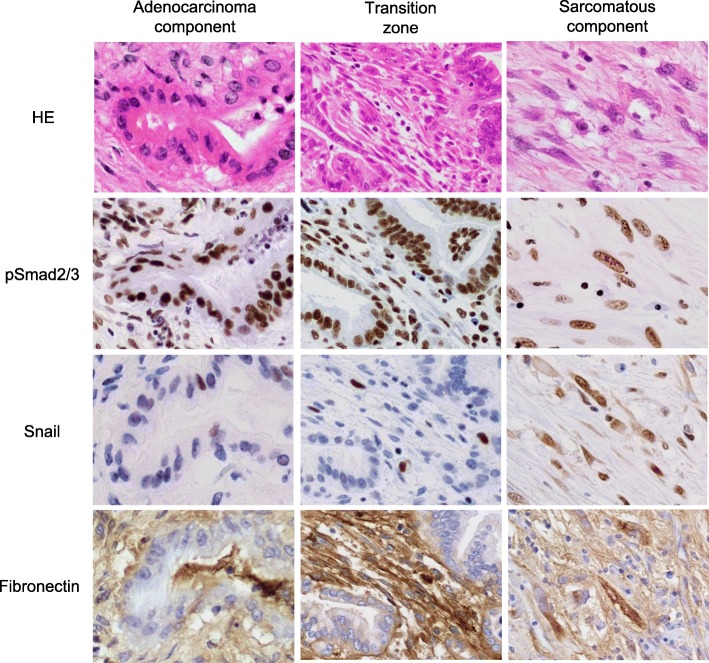
Immunohistochemical expression of EMT-related molecules. The expression of pSmad2/3 was observed in the nuclei of both the adenocarcinoma and sarcomatous components, whereas Snail and fibronectin were preferentially expressed in the sarcomatous component

The transition feature observed between the adenocarcinoma component and the sarcomatous component indicated the occurrence of EMT. To further clarify this, an immunohistochemical analysis was performed to examine the expression of pSmad2/3, Snail, and fibronectin. Within the tissue section, the expression of these molecules was separately evaluated in the adenocarcinoma component, the sarcomatous component, and the transition zone. As shown in Fig. 5, the expression of pSmad2/3 was observed in the nuclei of both the adenocarcinoma and the sarcomatous components, whereas Snail and fibronectin were preferentially expressed in the sarcomatous component.

A total of forty-four lymph nodes were resected, and histological examination showed the presence of carcinoma cells of the sarcomatous component in three lymph nodes around the tumor, where they were affected by the direct invasion into the lymph nodes, not by the metastasis via the lymphatic vessels. The final clinical stage was T3N1M0, stage IIB according to the UICC TNM staging system.

Following the operation, the patient completed a 6-month course of adjuvant chemotherapy with gemcitabine and was then followed up with CT. At 10 years after the operation, he is alive with no recurrence and treated with insulin for diabetes.

## Discussion

SCP is an extremely rare malignant tumor of the pancreas comprised of two independent components: ductal adenocarcinoma and sarcomatous tissues. The WHO classification of exocrine pancreatic tumors (5th edition, 2019) assigns SCP and carcinosarcoma of the pancreas (CSP) under the category of undifferentiated carcinomas [1]. According to the WHO classification (5th edition, 2019), SCP has cells with spindle cell morphology that may contain heterologous elements including bone and cartilage. Pathological diagnosis of SCP requires that at least 80% of the neoplasm displays spindle cell features, with or without heterologous differentiation. CSP is defined as a tumor composing of both epithelial and sarcomatoid elements, in which each component should constitute 30% of the neoplasm. Based on the WHO classification (5th edition, 2019), the present case was corresponded to SCP.

Although SCP and CSP have different pathologic features, both of them share similar clinical features and can be interpreted as more malignant variants of conventional pancreatic ductal adenocarcinoma [11]. In previously published reports, the terms of SPC and CSP have been often used interchangeably, and their definitions vary among the reports. In this context, literature review was conducted for CSP as well as SPC to clarify the clinical features of this rare type of tumor.

Based on the literatures concerning SCP [2–12] and CSP [16–32], they are more common in middle-aged and elderly people. The typical symptoms include nonspecific abdominal pain, weight loss, anorexia, nausea, and vomiting. When tumors are located in the pancreatic head, they cause early jaundice, which is commonly observed in other malignant neoplasms. They exhibit high rates of local recurrence and distant metastasis. The survival benefit of surgery remains uncertain, and no therapeutic strategies have been established. The prognosis is poor with a median overall survival time of 8 months and an average survival time of 9 months. An exceptional rare case has been documented in a 73-year-old female who underwent pancreatoduodenectomy and total gastrectomy, in which the patient survived for 15 years and 8 months after the operation, representing the longest survival time of SPC patients in the literature [9]. The patient in the present case is alive 10 years after the operation with no local recurrence and distant metastasis, and thus, it corresponds to the second longest survival time of SCP in the literature.

The mechanism underlying in the histogenesis of SCP can be explained by the transformation theory, where the sarcomatous component is derived from the adenocarcinoma component, and EMT is a plausible mechanism [3, 11]. In the present case, the immunohistochemical expression of pSmad2/3 in carcinoma cells could be regarded as an evidence that the intercellular signaling pathway of TGF-β was activated within the tumor. The expression of Snail and fibronectin in the sarcomatous cells further indicated the occurrence of EMT. Following EMT, the invasion of the carcinoma cells might have progressed, and the resultant sarcomatous cells formed a large tumor with an abundant fibrous stroma mediated by the local production of extracellular matrix proteins including fibronectin.

Although the exact mechanism explaining the patient’s long survival remains unclear, low malignant potential of the sarcomatous component might be related to the prolonged survival time in this case. The MIB-1 labeling index of the sarcomatous cells in SCP or CSP has been reported to be 50 to 90% [7, 29, 32]. In contrast, the MIB-1 labeling index of the sarcomatous component was 11% in this case. The result showed that the sarcomatoid cells had lower cell proliferative activity than those in previously reported SCP cases, which might lead to the reduced potential of local recurrence and metastasis, resulting in long-term survival. The small amount of adenocarcinoma component might also associate with the extension of the survival time.

Among undifferentiated carcinomas of the pancreas, it is reported that undifferentiated carcinoma with osteoclast-like giant cells tends to behave unexpectedly well and a substantial proportion of patients are alive after many years [33]. A recent study has shown that undifferentiated pancreatic carcinoma with osteoclast-like giant cells is enriched for PD-L1 expression, relative to conventional pancreatic ductal adenocarcinoma [34]. However, frequent expression of PD-L1 has been also observed in other types of undifferentiated pancreatic carcinomas; further studies on the significance of PD-L1 expression are necessary [35].

## Conclusions

The current case study presented a patient with SCP that was confirmed by pathological analysis including immunohistochemistry, and EMT was suggested to be involved in the histogenesis of SCP. Notably, the patient survived for 10 years after the operation, which corresponded to the second longest living individual with SCP reported in the English literature. Although the exact reason was not clear, the relatively low cell proliferative activity of sarcomatous cells might play a role in the prolonged survival of the patient.

## Data Availability

All data generated or analyzed during this study are included in this published article.

## Abbreviations

CSP: Carcinosarcoma of the pancreas
CT: Computed tomography
EMT: Epithelial-mesenchymal transition
MPD: Main pancreatic duct
MRCP: Magnetic resonance cholangiopancreatography
pSmad2/3: Phosphorylated Smad2/3
MRI: Magnetic resonance imaging
PD-L1: Programmed death-ligand 1
SCP: Sarcomatoid carcinoma of the pancreas
TGF-β: Transforming growth factor-β
UICC: Union for International Cancer Control
WHO: World Health Organization
